# Celebrating 10 Years of the National Comprehensive Cancer Control Program, 1998 to 2008

**Published:** 2009-09-15

**Authors:** Anne Major, Sherri L. Stewart

**Affiliations:** Division of Cancer Prevention and Control, Centers for Disease Control and Prevention; Centers for Disease Control and Prevention, Atlanta, Georgia

In 2008, the Division of Cancer Prevention and Control of the Centers for Disease Control and Prevention (CDC) celebrated the first 10 years of the National Comprehensive Cancer Control Program (NCCCP). This program was created by a group of public health professionals who recognized that a more collaborative approach was necessary to reduce the burden of cancer in the United States. They believed that coordination among the various sectors involved in cancer control would improve prevention, early detection, treatment, quality of care, and survival. We present a summary of how the movement began, the NCCCP's accomplishments, the program's collaboration with the National Partnership for Comprehensive Cancer Control, and current initiatives in the program. We also discuss the vision for the future of this program.

## A New Approach to Cancer Prevention and Control

After the beginning of the "war on cancer" in 1971 (1), there was a gradual buildup of cancer prevention, research, and treatment initiatives. By the end of the 1980s, however, the observable effect in reducing the incidence and mortality from cancer was less than had been anticipated. Even with the new research and program initiatives launched by CDC, the National Cancer Institute (NCI), and the American Cancer Society (ACS), it was unclear whether the goals set in the US Public Health Service's Healthy People initiative (2) and NCI's *Cancer Control Objectives for the Nation: 1985-2000* (3) would be met*.* What was needed was a more comprehensive and integrated approach that involved state agencies, local governments, private industry, professional organizations, volunteer organizations, the media, and other sectors affected by cancer (1).

In 1994, CDC, along with ACS, NCI, the Commission on Cancer of the American College of Surgeons, the North American Association of Central Cancer Registries, the Intercultural Cancer Council, the National Association of Chronic Disease Directors, and other public health leaders at state and national levels began promoting a comprehensive approach to cancer control. The approach coordinated and integrated cancer prevention and control programs across traditional funding boundaries. These organizations were later joined by C-Change (formerly the National Dialogue on Cancer) and the Lance Armstrong Foundation to become the National Partnership for Comprehensive Cancer Control (National Partners). A critical part of the success in developing and sustaining the new approach came from the timely and coordinated assistance of the National Partners.

From 1995 through 1998, CDC held meetings and workshops to gather input on the feasibility of implementing cancer control programs at the state level. CDC also conducted a baseline assessment of existing efforts and case studies of cancer control planning processes. From this effort, CDC published the initial definition and framework for comprehensive cancer control (CCC), a description of the essential elements, and a planning model (4).

In 1998, CDC began a pilot program that provided funding for 5 states and 1 tribal health board that had existing cancer control plans: Colorado, Massachusetts, Michigan, North Carolina, Texas, and the Northwest Portland Area Indian Health Board. This was the beginning of CDC's NCCCP. Miller et al describe these 6 programs in this issue of *Preventing Chronic Disease* (5).

## Ten Years of Success

By 2008, the number of programs participating in the NCCCP had increased to 65 (Figure). CDC awarded $22.4 million in fiscal year 2008 to 50 states, the District of Columbia, 7 tribal governments and organizations, and 7 territories and US-associated Pacific Islands jurisdictions to support the development and implementation of their CCC programs and plans. Part of this funding came from CDC's Division of Cancer Prevention and Control to provide base funding and technical assistance for CCC management, for cancer control planning and implementation, and for sustaining infrastructure. Additional CDC funding to NCCCP grantees supported specific colorectal, prostate, ovarian, and skin cancer control activities. Funds are not provided directly for cancer screening (6).

**Figure.**
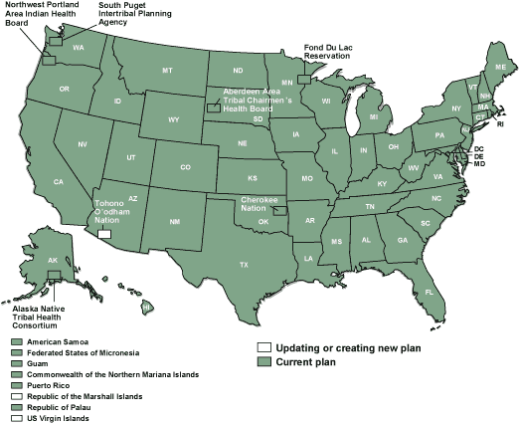
Status of cancer plans in 2008, National Comprehensive Cancer Control Program.

The NCCCP, in collaboration with the National Partners, supports the establishment and growth of state, tribal, territorial, and Pacific Islands cancer coalitions for the development of cancer plans. These coalitions comprise active representatives from state health departments, cancer treatment centers, and local cancer organizations and task forces. Each coalition reviews its existing cancer data to develop a plan for addressing cancer in that jurisdiction and identifying priorities. The coalition then uses evidence-based strategies and activities to implement the plan.

CDC worked closely with the initial programs to develop a framework for CCC, and through case studies of the programs published *Guidance for Comprehensive Cancer Control Planning, Volume 1: Guidelines* in 2002 (6). In 2007, CDC released *Comprehensive Cancer Control Promotional Toolkit* (7). This toolkit provides examples of presentations and other promotional information about CCC. With these additional resources and support, as well as with improved coordination, more coalitions were able to implement their plans. Many CCC coalitions have since secured private, state, tribal, territory, and local funding through various approaches, which include seeking and receiving status as a nonprofit organization. Those nonprofit coalitions work with their members to seek outside contributions to sustain efforts and pay for implementation of CCC strategies.

## Success in Partnership

In 2003, the CCC National Partners made site visits to deliver tailored technical assistance to CCC coalitions that needed help developing their activities and completing their cancer plans. From 2000 to 2007 the National Partners, in conjunction with ACS, conducted leadership institutes for teams of leaders from states, tribes, territories, and Pacific Islands jurisdictions to learn, share, and set strategic direction for their CCC initiatives. Altogether, thousands of CCC coalition members have attended the leadership institutes.

The National Partners also have developed resources for CCC coalitions. For example, the Cancer Control PLANET (Plan, Link, Act, Network with Evidence-based Tools [cancercontrolplanet.cancer.gov/]) Web portal was launched in April 2003 as an online data and planning tool to assist CCC practitioners. CancerPlan.org (https://www.cancerplan.org) is an online resource for CCC programs and coalitions that offers users real-time interaction with peers and enables sharing of ideas and resource materials. In May 2008, the National Partners, with leadership from C-Change, held the Comprehensive Cancer Control Policy and Practice Summit, which provided an opportunity for CCC coalition leaders and National Partners to identify and discuss key issues and corresponding policy solutions that have the potential to enhance their CCC efforts. This summit was the first time leaders from all states convened to discuss cross-state interests and ideas.

## Turning the Tide

During the past decade, 64 CCC programs have begun to implement the public health strategies in their cancer plans, and 1 has begun planning. To understand how specific issues are addressed and how to potentially measure the effect of the CCC approach, CDC conducted a review of the existing cancer plans in 2005 and published *Comprehensive Cancer Control Plans: A Content Review* (8). This review covered the 31 plans available by the end of 2004. Many programs documented the success of the comprehensive approach to collaboration for cancer control. For example, Minnesota publishes an annual report that describes major policy and prevention accomplishments and progress toward its incidence and mortality target objectives (9). The following are 2 examples of program success stories:

- Colorado: Under the banner of "Citizens for a Healthier Colorado," voluntary health organizations, tobacco control advocacy organizations, and statewide chronic disease coalitions including the Colorado Cancer Coalition advocated for an increase in the state's tobacco excise taxes. Of these new taxes, 16% would go to the prevention, early detection, and treatment of cancer, heart disease, and pulmonary diseases and 16% to tobacco use prevention. Since early 2005, $45 million has been distributed to support statewide and local efforts (10).
- Cherokee Nation: Cherokee Nation was the first tribal nation to develop a CCC plan for its population. In October 2006, Cherokee Nation successfully convened the first Cherokee Nation Cancer Summit to promote the Cherokee Nation CCC plan and its implementation and to increase awareness about cancer disparities among the community and its leaders, health professionals, and other entities. The release of the Cherokee Nation CCC plan at the summit increased credibility and awareness of the Cherokee Nation CCC plan and its goals. For example, as a result of the summit, the Cherokee Nation entered into a $1.5 million Memorandum of Understanding with Oklahoma University-Tulsa for cancer care and other chronic disease care. Cherokee Nation also is working with the Oklahoma Society of Clinical Oncology on an initiative to facilitate access to clinical trials in Oklahoma and surrounding states. The Cherokee Nation Web site will serve as the central site for information on this initiative (11).

The development of a comprehensive approach and the subsequent development of cancer plans and implementation of strategies has had a clear effect. Policy and systems approaches have improved. Some program success stories are described on the NCCCP Web site (10). Programs have also been encouraged by data showing that the incidence of and mortality for all cancers nationwide began to decline in 1999 (12).

## Vision of the Future

As the NCCCP matures and the results of CCC plan implementation are realized, CDC will continue to provide financial assistance, leadership, and guidance to CCC programs. In addition, CDC is engaged in separate coordinated research efforts to support the use of evidence-based practices in CCC programs to reduce cancer. Some specific examples of cancer surveillance research efforts include the publication of a comprehensive review of cancers associated with tobacco use in the United States (13). This review reported state-specific cancer incidence data for the 10 cancers for which there is convincing evidence of a direct causal relationship with tobacco use, according to the US Surgeon General. These data were widely disseminated among researchers, programs, and the public, through mailings, podcasts, media interviews, and distribution at program meetings. The data provide a baseline measure to monitor the effectiveness of tobacco and cancer control efforts aimed at reducing disease caused by tobacco use. With regard to health disparities, both CDC and CCC program staff contributed to a monograph describing cancer among the American Indian and Alaska Native population (14). Researchers analyzed data to present cancer incidence, risk factor, and screening information for different cancer sites (14). This information will be useful in the design and implementation of interventions to decrease cancer in this population. Plans are to develop a life-stage approach to cancer prevention to address upstream causes of cancer.

## Conclusion

During the last 10 years, a new movement has emerged in communities across the country to provide a comprehensive approach to cancer control. This new approach led to the formation of the NCCCP. CDC, in collaboration with the National Partners, works to build a sustainable direction for cancer prevention and control. As the NCCCP continues to move forward and work across all chronic disease programs, it will continue to support the best in partnership, program evaluation, and cancer control practice and to celebrate successes.
